# Supplementary material for: A Comprehensive Assessment of Ultraviolet-Radiation-Induced Mutations in Flammulina filiformis Using Whole-Genome Resequencing
Source: J Fungi (Basel). 2024 Mar 20;10(3):228. doi: 10.3390/jof10030228 (PMC10971301; doi:10.3390/jof10030228)
Supplement: Supplementary file 1 [file jof-10-00228-s001.zip › Supplementary Material S8/KEGG annotation/out/64381550635650.os/KO/out_map/map04912.html]

KEGG PATHWAY: GnRH signaling pathway - Reference pathway


|  |  |
| --- | --- |
| **GnRH signaling pathway - Reference pathway** |  |

[
Pathway menu
| Organism menu
| Pathway entry
| Show description
| User data mapping
]

Gonadotropin-releasing hormone (GnRH) secretion from the hypothalamus acts upon its receptor in the anterior pituitary to regulate the production and release of the gonadotropins, LH and FSH. The GnRHR is coupled to Gq/11 proteins to activate phospholipase C which transmits its signal to diacylglycerol (DAG) and inositol 1,4,5-trisphosphate (IP3). DAG activates the intracellular protein kinase C (PKC) pathway and IP3 stimulates release of intracellular calcium. In addition to the classical Gq/11, coupling of Gs is occasionally observed in a cell-specific fashion. Signaling downstream of protein kinase C (PKC) leads to transactivation of the epidermal growth factor (EGF) receptor and activation of mitogen-activated protein kinases (MAPKs), including extracellular-signal-regulated kinase (ERK), Jun N-terminal kinase (JNK) and p38 MAPK. Active MAPKs translocate to the nucleus, resulting in activation of transcription factors and rapid induction of early genes.

|  |  |
| --- | --- |
| Reference pathway | 100% |
